# Characterization of human Ccser2 as a protein tracking the plus-ends of microtubules

**DOI:** 10.1186/s13104-023-06475-z

**Published:** 2023-09-08

**Authors:** Yuko Shirai, Tomohiro Okuda, Kenzi Oshima, Daita Nadano

**Affiliations:** Department of Applied Biosciences, Graduate School of Bioagricultural Sciences, Nagoya University, Furo-cho, Chikusa, Nagoya, 464-8601 Japan

**Keywords:** Microtubules, Microtubule-associated proteins, Microtubule plus-end-tracking proteins, EB1, Breast cancer

## Abstract

**Objective:**

Microtubules, which are closely related to cell proliferation, have been the promising therapeutic target of cancer. Therefore, it is necessary to understand the intracellular control mechanisms of microtubules, the whole picture of which is still unclear though. Intracellular dynamics of microtubules are regulated by various microtubule-associated proteins, one group of which is microtubule plus-end-tracking proteins (+ TIPs), localizing to the extending tips of microtubules. Here, we report the identification and analysis of Ccser2 as a new + TIP in human breast cancer MCF-7 cells.

**Results:**

Ccser2 was found to be a member of + TIPs by microscopic observations including time-lapse imaging. The C-terminal region of Ccser2, including two SxIP motifs, was likely to be important for the tracking function. In MCF-7 cells, endogenous Ccser2 was mainly detected in the peripheral regions of microtubule fibers, suggesting that Ccser2 functions in cell projections.

**Supplementary Information:**

The online version contains supplementary material available at 10.1186/s13104-023-06475-z.

## Introduction

There were 2.3 million women diagnosed with breast cancer and 685,000 deaths globally in 2020 (from the website of the World Health Organization; Breast cancer. https://www.who.int/news-room/fact-sheets/detail/breast-cancer. Accessed 1 April 2023). A comprehensive understanding of the biology of normal mammary glands and breast cancer is necessary for the discovery of new therapies as well as prevention of the cancer. For example, the post-weaning phase of tissue regression in the mammary gland is called involution and has been reported to be associated with breast cancer [1]. We previously reported that Ccser2 (formerly Gcap14) was expressed in the involuting mouse mammary gland at the mRNA level, was specifically associated with microtubules (MTs) in cells, and exhibited MT-bundling ability in vitro [2]. Based on molecular links between MTs and breast cancer [3], we further sought to analyze Ccser2 expressed in a human breast cancer cell line, MCF-7.

## Main text

### Materials and methods

Mammalian cell culture, RNA preparation from culture cells, and reverse transcription-PCR have been described previously [4]. The cDNA encoding the full-length Ccser2 (accession no. XP_006717957.1) was amplified by PCR using PrimeSTAR HS DNA polymerase (Takara, Japan) and MCF-7 cDNA as follows. Primers: 5′-TTTGATATCATGCTTGATGTGGATCTGCC-3′ (forward, EcoRV site underlined) and 5′-TTTGTCGACTTAATGTATCTTTGGTTTAGG-3′ (reverse, SalI site underlined). PCR was carried by denaturation at 95 °C for 4 min followed by 30 cycles of denaturation at 98 °C for 10 s, annealing at 55 °C for 5 s, and an extension at 72 °C for 3 min. The full-length cDNA of human Ccser2 was cloned into the pCMV-Tag2B vector for the expression N-terminally FLAG-tagged Ccser2 in mammalian cells. The cDNA was also cloned into the pEGFP-C1 vector for the expression of N-terminally green fluorescent protein (GFP)-fused Ccser2. Deletion mutant preparation was performed as described previously [4]. The cDNA of human EB1 was cloned into the pEF1/mKO-Myc-His vector for the expression of C-terminally His-tagged (6 × histidine-tagged) EB1. Transient transfection of MCF-7 cells was performed by using PEI-Max as described previously [4]. Vector DNA (3 μg) was introduced into cells seeded at 3 × 10^5^/dish in a 35-mm culture dish.

Immunoblotting, immunoprecipitation under MT-depolymerizing conditions, and immunofluorescent analysis of fixed culture cells have been described in our previous reports [2, 5]. Rabbit anti-human Ccser2 antibody was raised against thioredoxin fused to a peptide (residues 222–493) of the protein. The following commercial antibodies were used: mouse antibodies against a FLAG tag and α-tubulin (Sigma-Aldrich), rabbit anti-GFP antibody (MBL, Japan), and mouse anti-6 × histidine antibody (FUJIFILM Wako Pure Chemicals, Japan). For antibody absorption, the antigen of anti-human Ccser2 antibody was immobilized on CNBr-activated Sepharose 4B (Cytiva), mixed with the antibody, and rotated at 4 °C for 2 h. The solution was then collected by filtration and used as the absorbed antibody. In the immunoblotting of cell lysates, proteins from about 3 × 10^4^ cells were loaded into each well of the gel. In immunoprecipitation, the protein content of cell lysates was determined by the BCA protein assay reagent (Pierce). Aliquots of the lysates containing 1.25 mg protein each were subjected to the analysis. For the immunocytochemical localization of Ccser2 in MCF-7 cells, cells were grown on coverslips, plunged into − 20 °C methanol (100%), and kept at − 20 °C for 10 min [6]. After blocking with phosphate-buffed saline containing 10% normal goat serum (Sigma-Aldrich) and 1% bovine serum albumin, cells were incubated with antibodies against human Ccser2 and α-tubulin at room temperature for 60 min. After rinsing with phosphate-buffered saline containing 0.1% Tween 20, cells were incubated with appropriate fluorophore-conjugated secondary antibodies at room temperature for 30 min. Cell fixation with glutaraldehyde was also performed according to the previous report [7]. Time-lapse imaging of fluorescent protein localization was performed on a confocal laser microscope (Fluoview FV1000D, Olympus, Japan). Just before the microscopic observation, cells plated on a coverslip were transferred to a culture dish containing DMEM with HEPES (FUJIFILM Wako Pure Chemicals) and placed in a stage incubator to maintain them at 37 °C. Images were acquired at 5-s intervals.

## Results and discussion

Intracellular dynamics of MTs are regulated by various MT-associated proteins. Mouse Ccser2 was an MT-associated protein [2]. To examine the function in its human counterpart, the cDNA of human Ccser2 from MCF-7 cells was cloned into the expression vectors and transiently introduced into MCF-7 cells. Ccser2 was shown to be co-localized with MTs (Additional file 1: Fig. S1a). In case of the high expression (i.e., bright cells under a fluorescence microscope with low magnification), human Ccser2 decorated MTs uniformly, and MTs were reorganized and became thicker (Additional file 1: Fig. S1b). These data were essentially the same as observed in cells transiently overexpressing mouse Ccser2 [2].

**Fig. 1:**
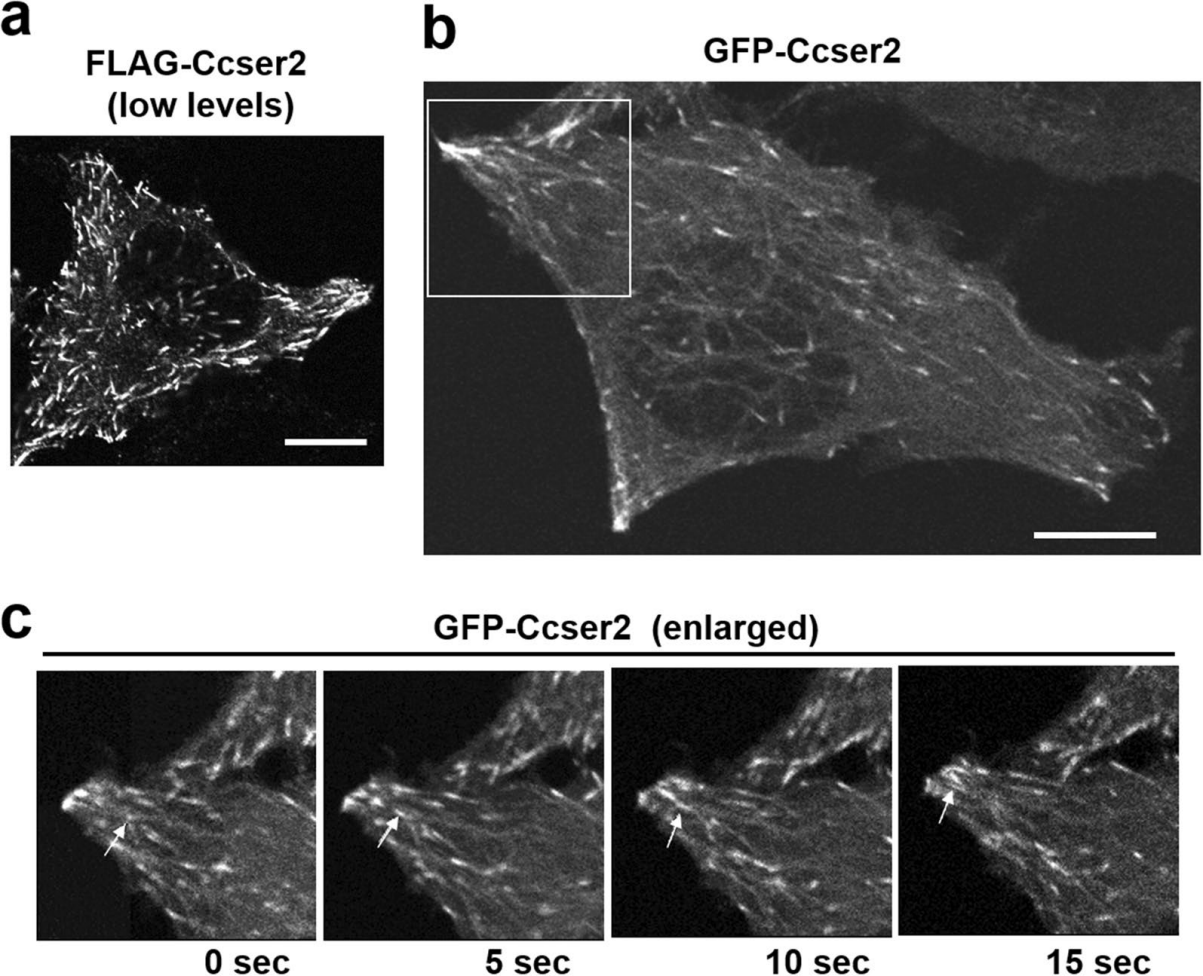
Identification of human Ccser2 as a + TIP. **a** MCF-7 cells, transiently expressing FLAG-Ccser2 at low levels, were fixed with cold methanol, subjected to immunostaining with anti-FLAG antibody, and observed by confocal microscopy. **b** Time-lapse microscopy of one of the MCF-7 cells expressing GFP-Ccser2 (see Additional file 2: Movie S1). Bars, 10 μm. **c** Time series of the boxed area in **b** are enlarged. GFP-Ccser2 moving like a comet is indicated by the arrows

However, in the transfected cells where the expression of human Ccser2 seemed relatively low (faintly fluorescent cells under a fluorescence microscope with low magnification), Ccser2 exhibited dot-like structures that were attached to apparent MT ends (Fig. 1a). The image was similar to that of intracellular MT plus-end-tracking proteins (+ TIPs). + TIPs are a group of MT-associated proteins and localize to extending MT tips [3]. Live imaging of the cells expressing GFP-Ccser2 at low levels showed that this protein was moving to the cell periphery forming a comet-like structure [8] (Fig. 1b, c and Additional file 2: Movie S1), supporting Ccser2 as a new + TIP.

**Fig. 2:**
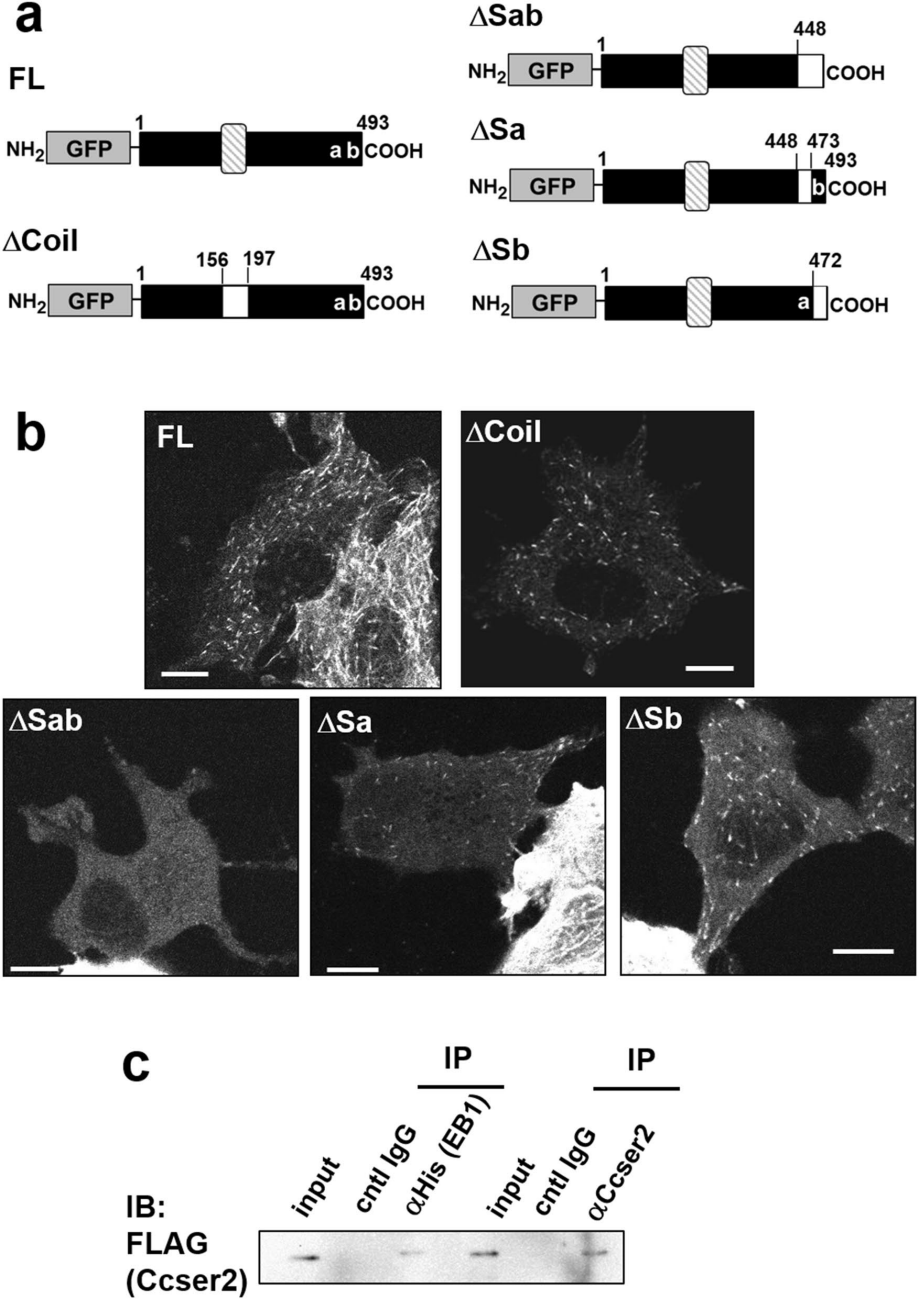
Human Ccser2 belongs to SxIP proteins. **a** Schematic representation of the structures of full-length Ccser2 (FL) and its deletion mutants prepared in this study. The slashed boxes indicate the predicted coiled-coil region. The “a” and “b” indicate the SxIP motifs. The open boxes indicate deleted regions. **b** Microscopic observation of MCF-7 cells transiently expressing the proteins indicated in **a**. Bars, 10 μm. **c** Co-immunoprecipitation of Ccser2 and EB1. MCF-7 cells were co-transfected with the expression vectors of FLAG-Ccser2 and EB1-His and subjected to immunoprecipitation (IP) with anti-Ccser2 and anti-His tag. Each immune complex was analyzed by immunoblotting (IB) with anti-FLAG antibody. Eight percent of the starting cell lysate (indicated as “input”) was loaded as control. A full-size uncropped image of the immunoblot is included in Additional file 4: Figure S3

We then investigated the mechanisms underlying the tracking function. Ccser2 shows no significant homology to known + TIPs [9] in amino acid sequences. However, this protein in humans as well as in mice (Additional file 3: Fig. S2) has two kinds of potentially functional regions (Fig. 2a): (i) a coiled-coil region in the center, which has been indicated by the software, Coiled-coil prediction [10] and (ii) two SxIP motifs (consensus, (S/T)-x-(I/L)-P), which are required for MT association of a group of + TIPs, or SxIP proteins [11]. As for the latter, it has been reported that, in addition to the SxIP sequence, the surrounding amino acid residues are also included in the association [11, 12]. To determine the contribution of these regions to the tracking function, the coiled-coil region was first deleted. However, this did not affect co-localization between this protein and MTs (Fig. 2b, panel ∆Coil). This region might be utilized for interactions between + TIPs at growing MT ends. Next, the two regions containing the SxIP motifs were examined. When both regions were removed, the deletion mutant was no longer bound to MTs and uniformly present in the cytosol (Fig. 2b, panel ∆Sab). When one of the two regions was deleted, some comets were observed, albeit reduced (Fig. 2b, panels ∆Sa and ∆Sb), indicating that both regions support the + TIP function of Ccser2.

Many + TIPs, including SxIP proteins, can associate with the elongating ends of MTs by binding to the hub protein EB1 [9, 12]. SxIP proteins bind to EB1 through the SxIP motif, which binds to the C terminal helix bundle of EB1 homodimer [11]. The interaction between Ccser2 and EB1 in the cells overexpressing these two proteins was tested by immunoprecipitation under MT-depolymerizing conditions. Co-sedimentation of Ccser2 and EB1 supports that Ccser2 belongs to SxIP proteins (Fig. 2c).

**Fig. 3:**
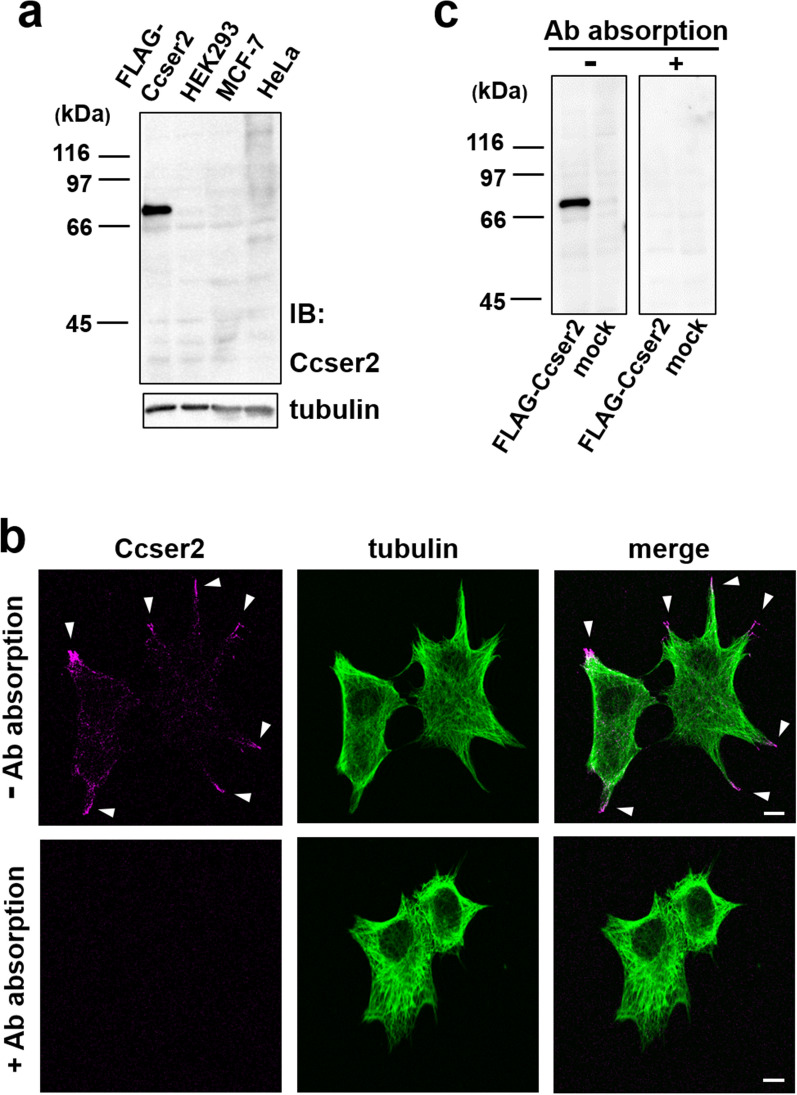
Detection of endogenous Ccser2 in MCF-7 cells. **a** Whole lysates of three cancer cell lines, HEK293, MCF-7, and HeLa, and of MCF-7 cells transfected with the FLAG-Ccser2 expression vector were subjected to immunoblotting with anti-human Ccser2 antibody (upper blot). The same blot was stripped and re-proved with anti-α-tubulin antibody (lower blot). Full-size uncropped images of the immunoblots are included in Additional file 5: Figure S4 (upper blot) and Additional file 6: Figure S5 (lower blot). **b** MCF-7 cells were fixed with cold methanol, subjected to double-immunostaining with anti-human Ccser2 and anti-α-tubulin, and observed by confocal microscopy. The arrowheads point to clusters of Ccser2. Bars, 10 μm. The antibody (Ab) absorption was confirmed by immunoblotting in **c**. **c** Two identical blots of the lysates of MCF-7 cells transfected with the FLAG-Ccser2 expression vector and pCMV-Tag2B (mock) were prepared by SDS-PAGE and electroblotting at the same time. One was stained with anti-Ccser2 antibody (left blot). After the treatment of the antibody with its immunogen, the other was stained with the treated antibody (right blot). Full-size uncropped images of the immunoblots are included in Additional file 7: Figure S6 (left blot) and Additional file 8: Figure S7 (right blot)

To find clues to the role of Ccser2 in cancer cells, we tried to observe endogenous Ccser2 in MCF-7 cells. The expression of Ccser2 at the protein level was examined by immunoblotting with the antibody against human Ccser2. Unfortunately, no prominent band was detected in the three cell lines, including MCF-7 (Fig. 3a). The same result was obtained in immunoblotting with loading more amount of the total proteins (Additional file 9: Fig. S8). Other isoforms of Ccser2, which are considered to be generated by alternative splicing, are listed in the public gene database (Gene ID, 54462; NCBI Gene. https://www.ncbi.nlm.nih.gov/gene. Accessed 1 April 2023). However, these results did not indicate their presence. In general, the expression levels of individual + TIPs are much lower than the hub protein, EB1 [12]. It seems that a small amount of Ccser2 exists at the level of whole cells and works locally on MTs.

The intracellular localization of endogenous Ccser2 was then observed by fluorescence microscopy. Fluorescent signals of Ccser2 in MCF-7 were reproducibly detected on MTs at the cell periphery (Fig. 3b, upper images). The peripheral region is densely populated with proteins, including cytoskeletal proteins, suggesting the possibility of nonspecific signals. This was unlikely because the signals disappeared after the pre-absorption of the anti-Ccser2 antibody with its immunogen (Fig. 3b, lower images; the complete absorption was confirmed by immunoblotting in Fig. 3c). These data suggest that endogenous Ccser2 functions in cell projections. EB1 and various + TIPs are present at the plus ends of MTs and constitute distinct multivalent interactions to function for a specific task [11, 12]. + TIPs in cell projections are involved in MT reorganization and epithelial remodeling [3, 13]. Ccser2, together with other + TIPs, may have a modulatory role in these phenomena. Microfilaments as well as MTs are involved in cell migration, which is important for tissue remodeling [14]. Methanol fixation, which was used for immunocytochemical observation of Ccser2, is not suitable for microfilament labeling with phalloidin [15]. To examine the co-localization of endogenous Ccser2 and microfilaments in MCF-7 cells, we attempted to observe Ccser2 using glutaraldehyde fixation, which is considered optimal for the maximum preservation of cytoskeletal structures [5–7]. However, Ccser2 could not be detected clearly after this fixation, even under overexpression conditions (Additional file 13: Fig. S12).

In conclusion, a + TIP expressed in breast cancer cells was found, and its tracking mechanism was uncovered. Specifically, (1) the properties of Ccser2 as a + TIP were identified by the live imaging, (2) two functional SxIP motifs were found to be included in this protein, and (3) endogenous Ccser2 was observed in cell projections in MCF-7 cells. While EB1, a representative + TIP, has been reported to be highly expressed in a variety of cancers [12], the relationship between the dozens of + TIPs and cancer is still largely unknown [3]. It is expected that further research on Ccser2 and its interaction with other + TIPs will lead to new insights into MT control mechanisms and cancer therapy.

## Limitations

A limitation of our studies is that they were conducted mainly in a breast cancer cell line, MCF-7. It would be important to confirm these findings in other breast cancer cells. Ccser2 has been suggested to be expressed in many cancer cell lines originating from various tissues (CCSER2 in The Human Protein Atlas. https://www.proteinatlas.org. Accessed 1 April 2023). The interactors of Ccser2, MTs, and EB1, are ubiquitously expressed. It would be interesting to investigate Ccser2 in other malignant tumor cells.

Since Ccser2 is correlated with the mouse mammary gland at the mRNA level, it is important to check the expression of Ccser2 in other cell types of the mammary gland, such as glandular epithelial cells, macrophages, and foam cells.

Most of the present characterization, including co-localization of Ccser2 with MTs in the live imaging and its identification as one of the SxIP proteins, was performed under overexpression conditions. Hence, the data could be the artifact of overexpression.

### Supplementary Information

**Additional file 1: Figure S1.** Fluorescence imaging of MCF-7 cells expressing human Ccser2. The vector for the expression of FLAG-Ccser2 (**a**), GFP-Ccser2 (**b**), or GFP (**c**) was transiently transfected into MCF-7 cells. In (**a**), the transfected cells were fixed with cold methanol, double-stained with anti-FLAG and anti-α-tubulin, and observed by confocal microscopy. In (**b**) and (**c**), the living transfected cells were observed by confocal microscopy. Bars, 10 μm.

**Additional file 2: Movie S1.** Behavior of GFP-Ccser2 in a living MCF-7 cell. MCF-7 cells expressing GFP-Ccser2 at low levels were imaged with a 5 s interval using Fluoview FV1000D. The image sequence corresponds to Figure 1b, c.

**Additional file 3: Figure S2. **Amino acid sequence alignment of human Ccser2 (upper) and mouse Ccser2 (lower). Numbers indicate positions of the amino acids. Vertical lines represent identity between a corresponding pair of amino acid residues, and dots represent similarity. Peptide sequences corresponding to a predicted coiled-coil region (Coil) and two SxIP motifs (Sa and Sb) are indicated by the thick horizontal bars.

**Additional file 4:** **Figure S3.** Full-size uncropped image of the immunoblot presented in Figure 2c. The red box indicates the bands in the figure; FLAG-Ccser2.

**Additional file 5:** **Figure S4.** Full-size uncropped image of the immunoblot presented in Figure 3a (upper blot). The red box indicates where the image was cropped to be presented in the figure. The band of FLAG-Ccser2 in the far-left lane is indicated by the arrow.

**Additional file 6:** **Figure S5.** Full-size uncropped image of the immunoblot presented in Figure 3a (lower blot). The red box indicates the bands in the figure; α-tubulin.

**Additional file 7:** **Figure S6.** Full-size uncropped image of the immunoblot presented in Figure 3c (left blot). The red box indicates where the image was cropped to be presented in the figure. The band of FLAG-Ccser2 in the left lane is indicated by the arrow.

**Additional file 8:** **Figure S7.** Full-size uncropped image of the immunoblot presented in Figure 3c (right blot). The red box indicates where the image was cropped to be presented in the figure.

**Additional file 9:** **Figure S8.** Immunoblotting of the lysates of MCF-7 cells containing large amounts of total proteins. Detection of the same blot with anti-human Ccser2 antibody was performed with a short exposure (left blot) and with a long exposure (middle blot). This blot was then stripped and re-proved with anti-α-tubulin antibody (right blot). The following cell lysates were loaded onto the gel: MCF-7 expressing human FLAG-human Ccser2 at low levels (lane 1); 3, 6, and 12 x 10^4^ cells of MCF-7 (lanes 2, 3, and 4, respectively); MCF-7 expressing GFP-human Ccser2 (lane 5). The band of FLAG-Ccser2 (low levels) in lane 1 is indicated by the asterisk. Full-size uncropped images of the immunoblots are included in Additional file 10: Figure S9 (left blot); Additional file 11: Figure S10 (middle blot); and Additional file 12: Figure S11 (right blot).

**Additional file 10:** **Figure S9.** Full-size uncropped image of the immunoblot presented in Figure S8 (left blot). The red box indicates where the image was cropped to be presented in the figure. The band of GFP-Ccser2 in lane 5 is indicated by the arrow.

**Additional file 11:** **Figure S10.** Full-size uncropped image of the immunoblot presented in Figure S8 (middle blot). The red box indicates where the image was cropped to be presented in the figure. The band of FLAG-Ccser2 (low levels) in lane 1 is indicated by the arrow.

**Additional file 12:** **Figure S11.** Full-size uncropped image of the immunoblot presented in Figure S8 (right blot). The red box indicates the bands in the figure; α-tubulin.

**Additional file 13:** **Figure S12. **Immunocytochemical detection of human Ccser2 after glutaraldehyde fixation. MCF-7 cells (top panels) and MCF-7 cells transiently expressing FLAG-human Ccser2 (middle and bottom panels) were fixed with glutaraldehyde (top and middle panels) or cold methanol (bottom panels). These fixed cells were then subjected to double-immunostaining with antibodies against human Ccser2 and α-tubulin. Bars, 10 μm.

## Data Availability

The data underlying this article are available from the corresponding author upon request.

## Abbreviations

GFP: Green fluorescent protein
MT: Microtubule
+ TIP: Microtubule plus-end-tracking protein
