# Supplementary figures and images for: Characterization of human Ccser2 as a protein tracking the plus-ends of microtubules
Source: BMC Res Notes. 2023 Sep 8;16:198. doi: 10.1186/s13104-023-06475-z (PMC10486078; doi:10.1186/s13104-023-06475-z)

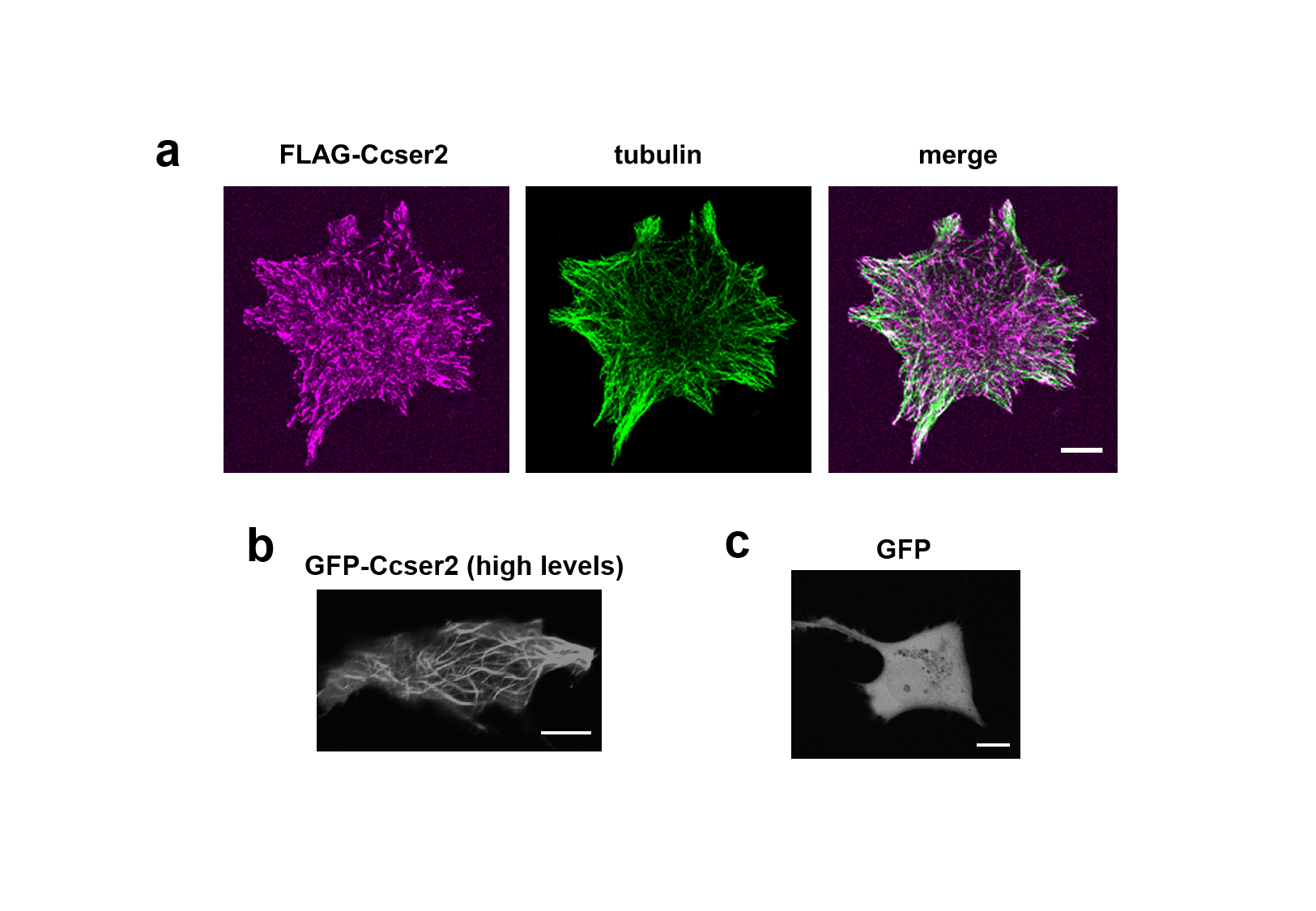

Supplement: Supplementary file 1 — Additional file 1: Figure S1. Fluorescence imaging of MCF-7 cells expressing human Ccser2. The vector for the expression of FLAG-Ccser2 (a), GFP-Ccser2 (b), or GFP (c) was transiently transfected into MCF-7 cells. In (a), the transfected cells were fixed with cold methanol, double-stained with anti-FLAG and anti-α-tubulin, and observed by confocal microscopy. In (b) and (c), the living transfected cells were observed by confocal microscopy. Bars, 10 μm. [file 13104_2023_6475_MOESM1_ESM.tif]

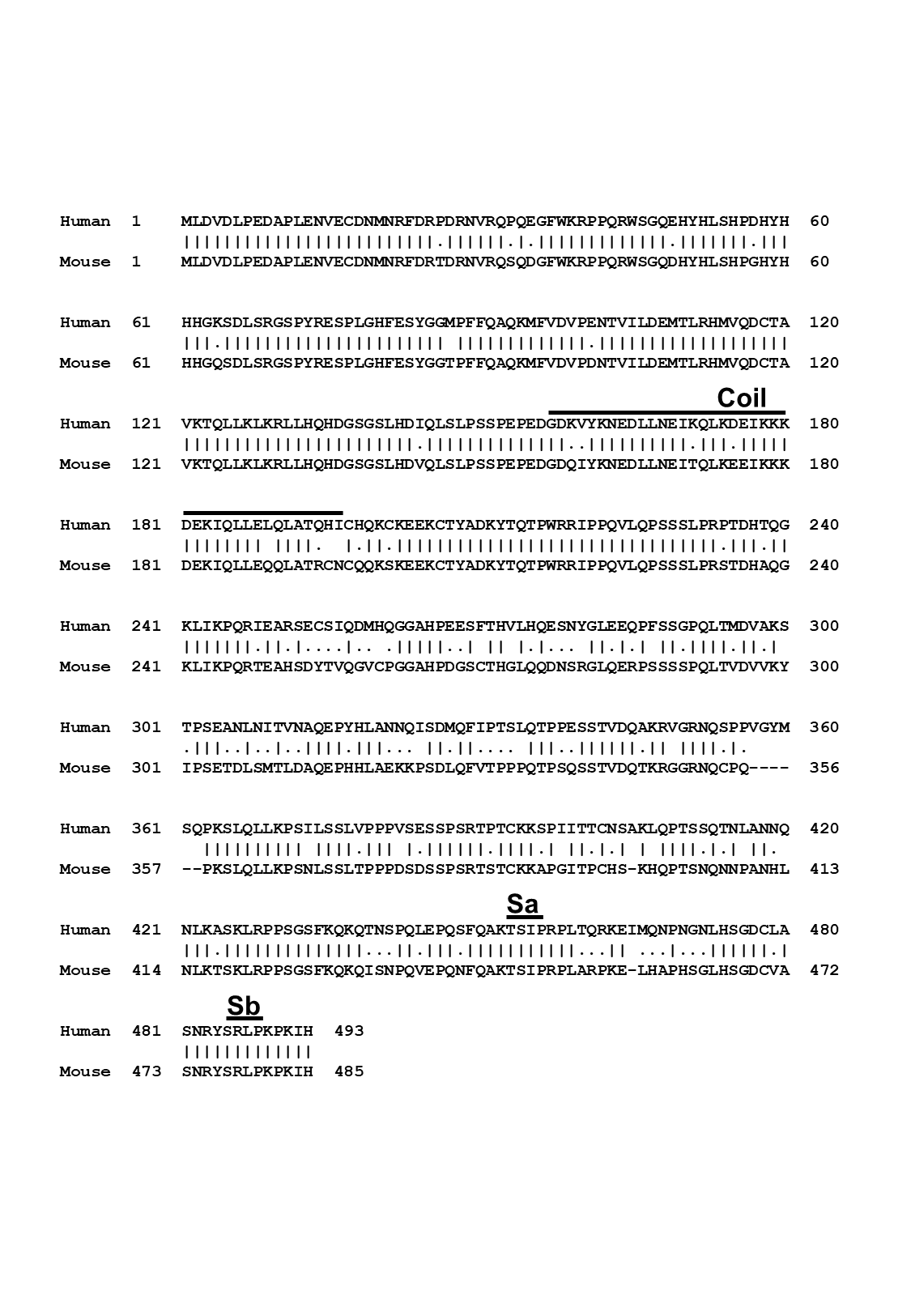

Supplement: Supplementary file 3 — Additional file 3: Figure S2. Amino acid sequence alignment of human Ccser2 (upper) and mouse Ccser2 (lower). Numbers indicate positions of the amino acids. Vertical lines represent identity between a corresponding pair of amino acid residues, and dots represent similarity. Peptide sequences corresponding to a predicted coiled-coil region (Coil) and two SxIP motifs (Sa and Sb) are indicated by the thick horizontal bars. [file 13104_2023_6475_MOESM3_ESM.tif]

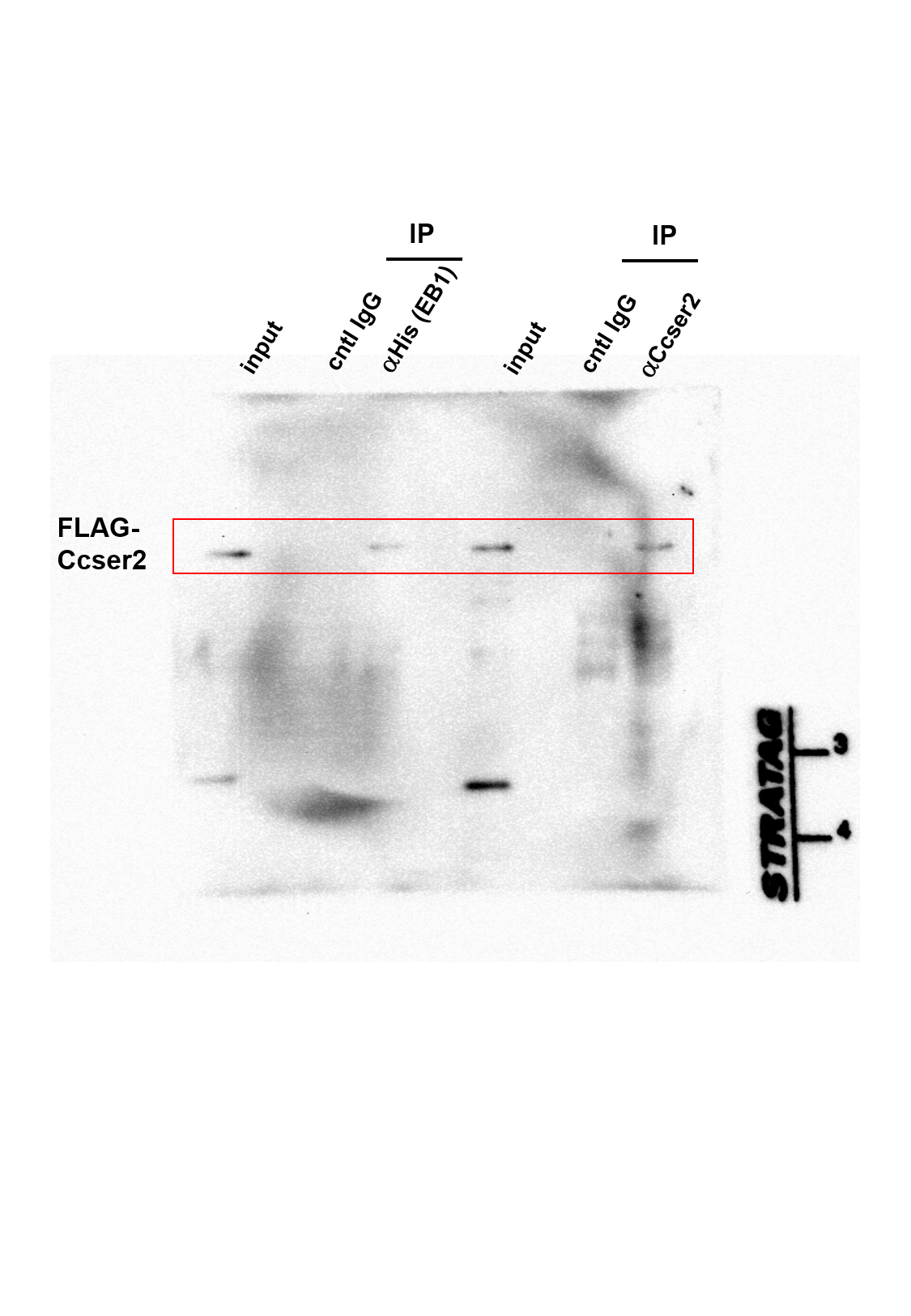

Supplement: Supplementary file 4 — Additional file 4: Figure S3. Full-size uncropped image of the immunoblot presented in Figure 2c. The red box indicates the bands in the figure; FLAG-Ccser2. [file 13104_2023_6475_MOESM4_ESM.tif]

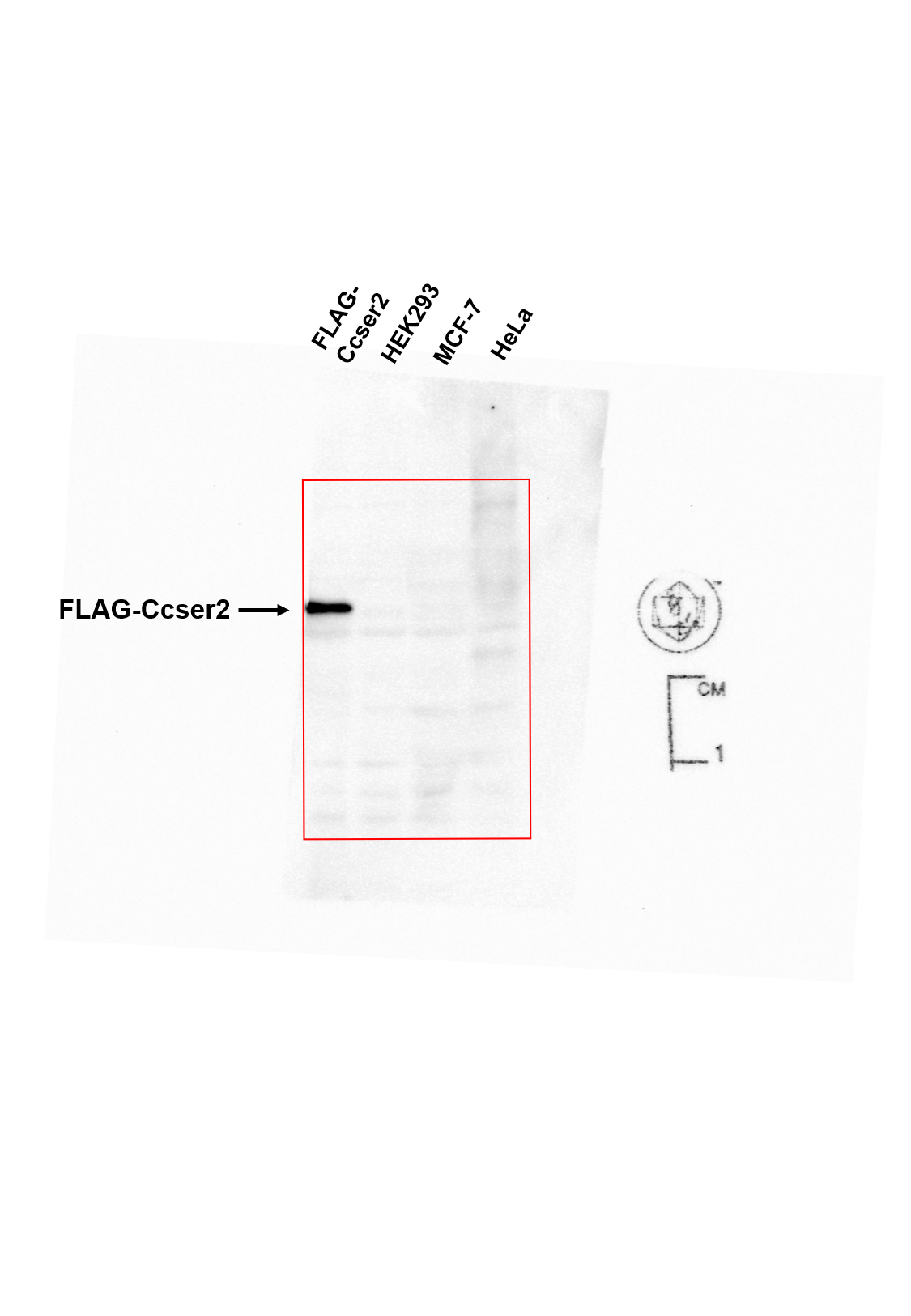

Supplement: Supplementary file 5 — Additional file 5: Figure S4. Full-size uncropped image of the immunoblot presented in Figure 3a (upper blot). The red box indicates where the image was cropped to be presented in the figure. The band of FLAG-Ccser2 in the far-left lane is indicated by the arrow. [file 13104_2023_6475_MOESM5_ESM.tif]

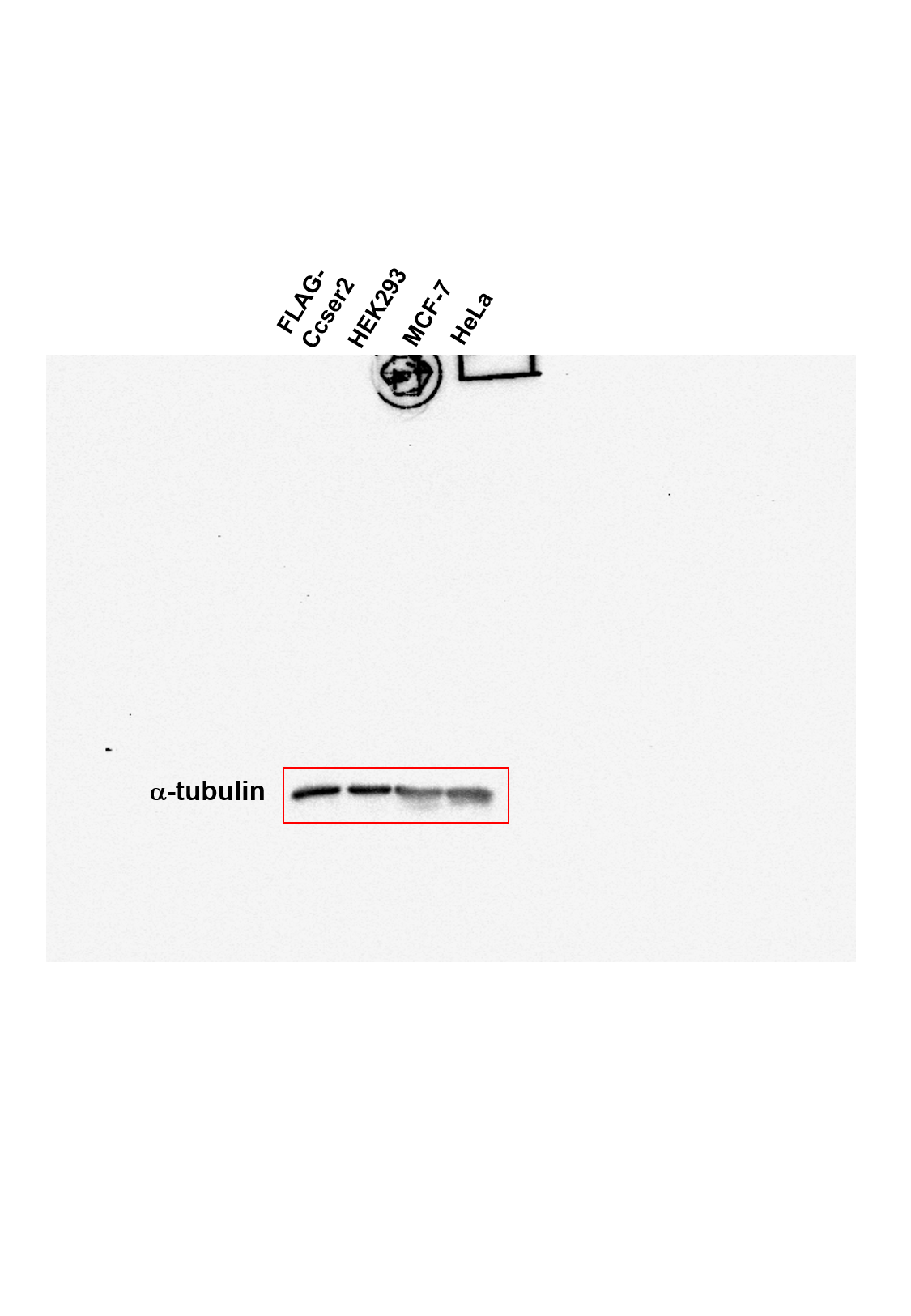

Supplement: Supplementary file 6 — Additional file 6: Figure S5. Full-size uncropped image of the immunoblot presented in Figure 3a (lower blot). The red box indicates the bands in the figure; α-tubulin. [file 13104_2023_6475_MOESM6_ESM.tif]

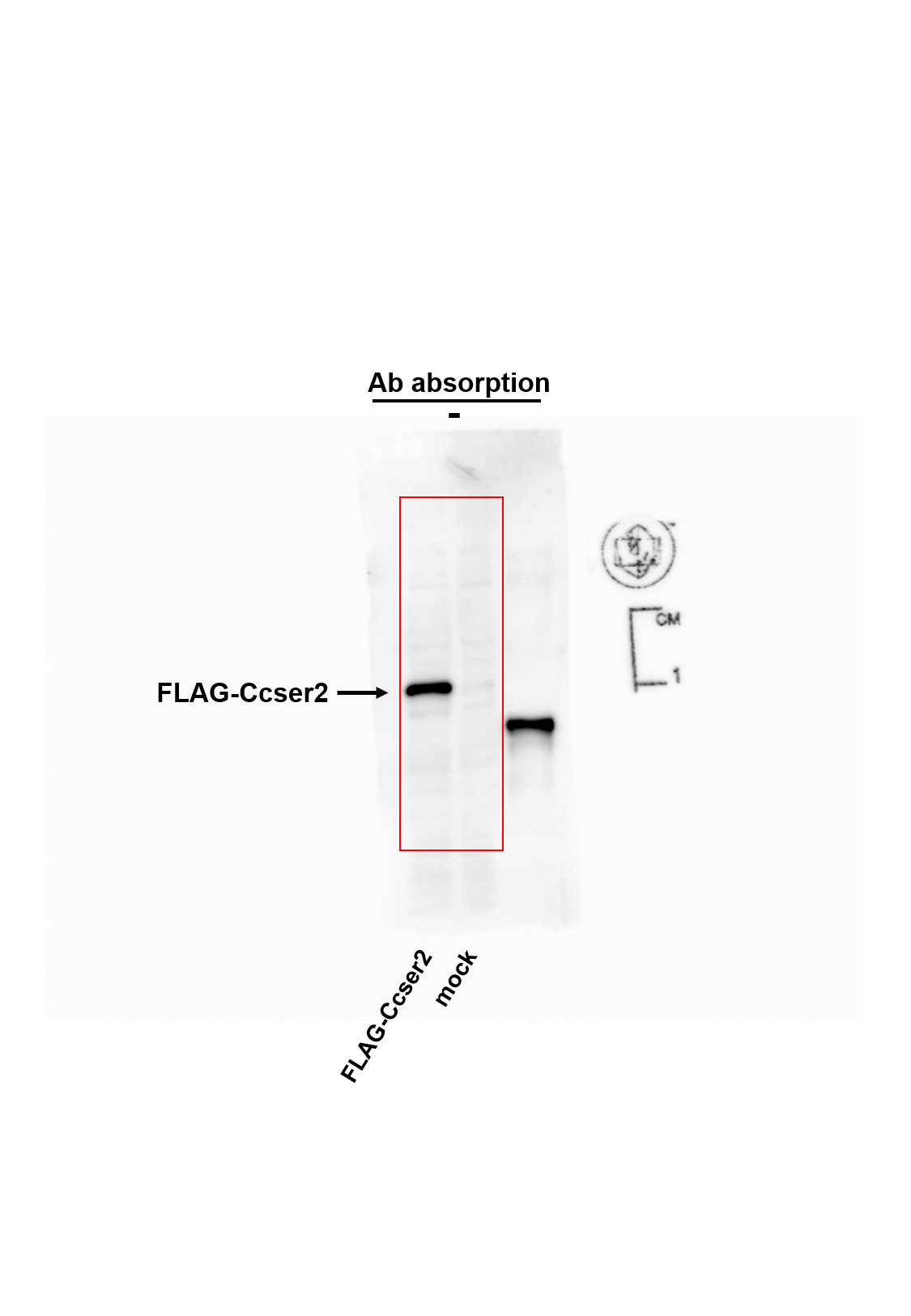

Supplement: Supplementary file 7 — Additional file 7: Figure S6. Full-size uncropped image of the immunoblot presented in Figure 3c (left blot). The red box indicates where the image was cropped to be presented in the figure. The band of FLAG-Ccser2 in the left lane is indicated by the arrow. [file 13104_2023_6475_MOESM7_ESM.tif]

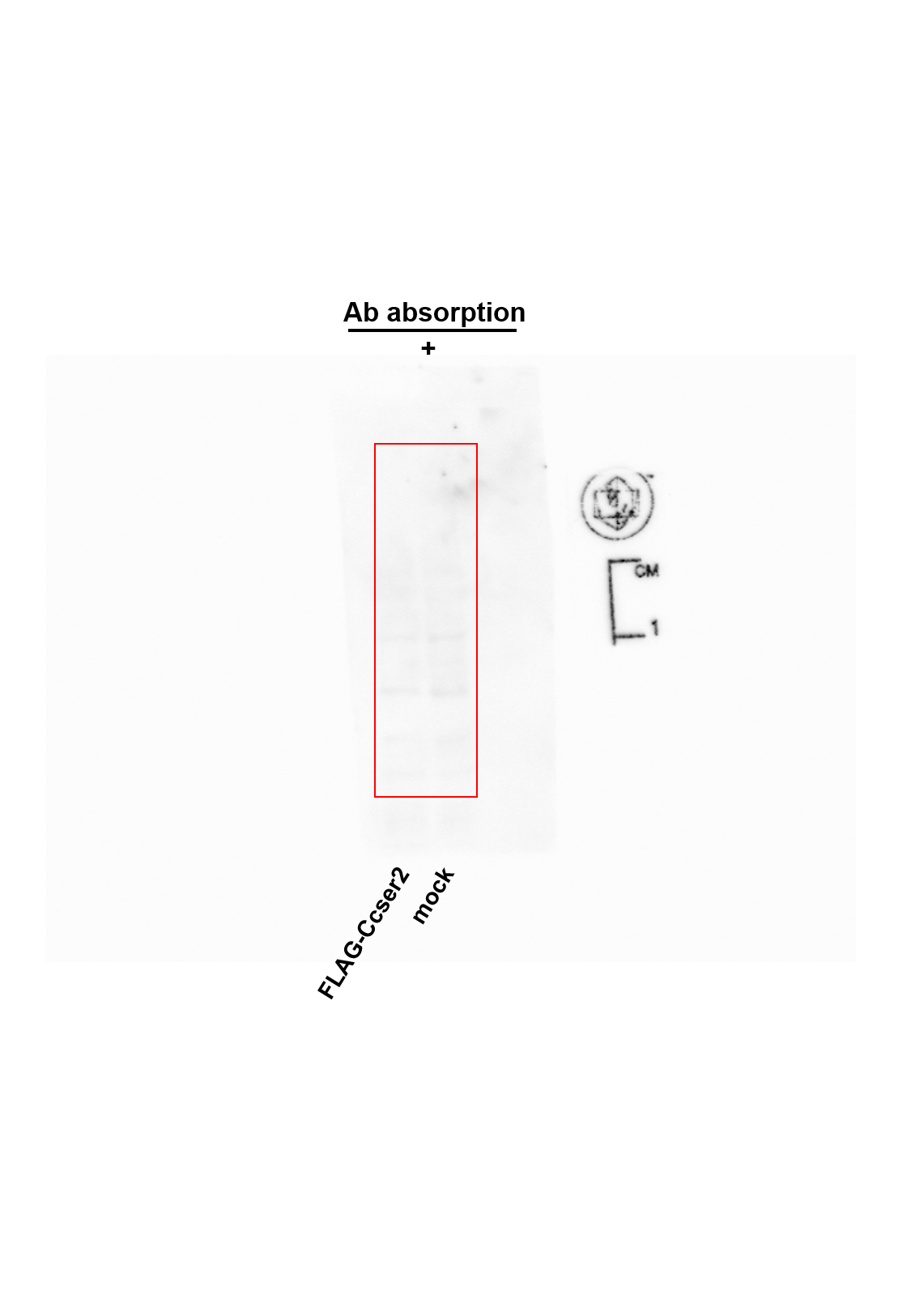

Supplement: Supplementary file 8 — Additional file 8: Figure S7. Full-size uncropped image of the immunoblot presented in Figure 3c (right blot). The red box indicates where the image was cropped to be presented in the figure. [file 13104_2023_6475_MOESM8_ESM.tif]

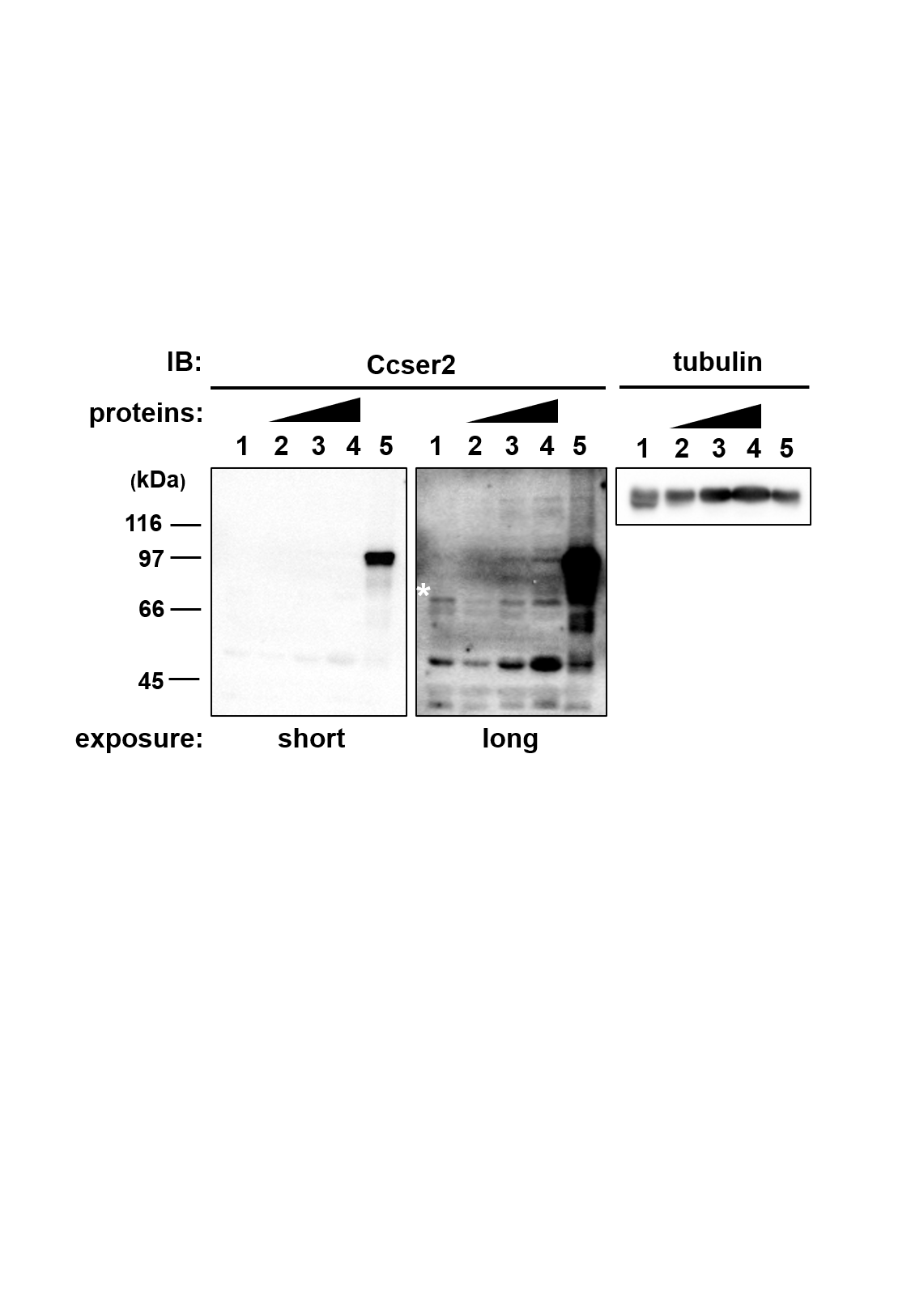

Supplement: Supplementary file 9 — Additional file 9: Figure S8. Immunoblotting of the lysates of MCF-7 cells containing large amounts of total proteins. Detection of the same blot with anti-human Ccser2 antibody was performed with a short exposure (left blot) and with a long exposure (middle blot). This blot was then stripped and re-proved with anti-α-tubulin antibody (right blot). The following cell lysates were loaded onto the gel: MCF-7 expressing human FLAG-human Ccser2 at low levels (lane 1); 3, 6, and 12 x 104 cells of MCF-7 (lanes 2, 3, and 4, respectively); MCF-7 expressing GFP-human Ccser2 (lane 5). The band of FLAG-Ccser2 (low levels) in lane 1 is indicated by the asterisk. Full-size uncropped images of the immunoblots are included in Additional file 10: Figure S9 (left blot); Additional file 11: Figure S10 (middle blot); and Additional file 12: Figure S11 (right blot). [file 13104_2023_6475_MOESM9_ESM.tif]

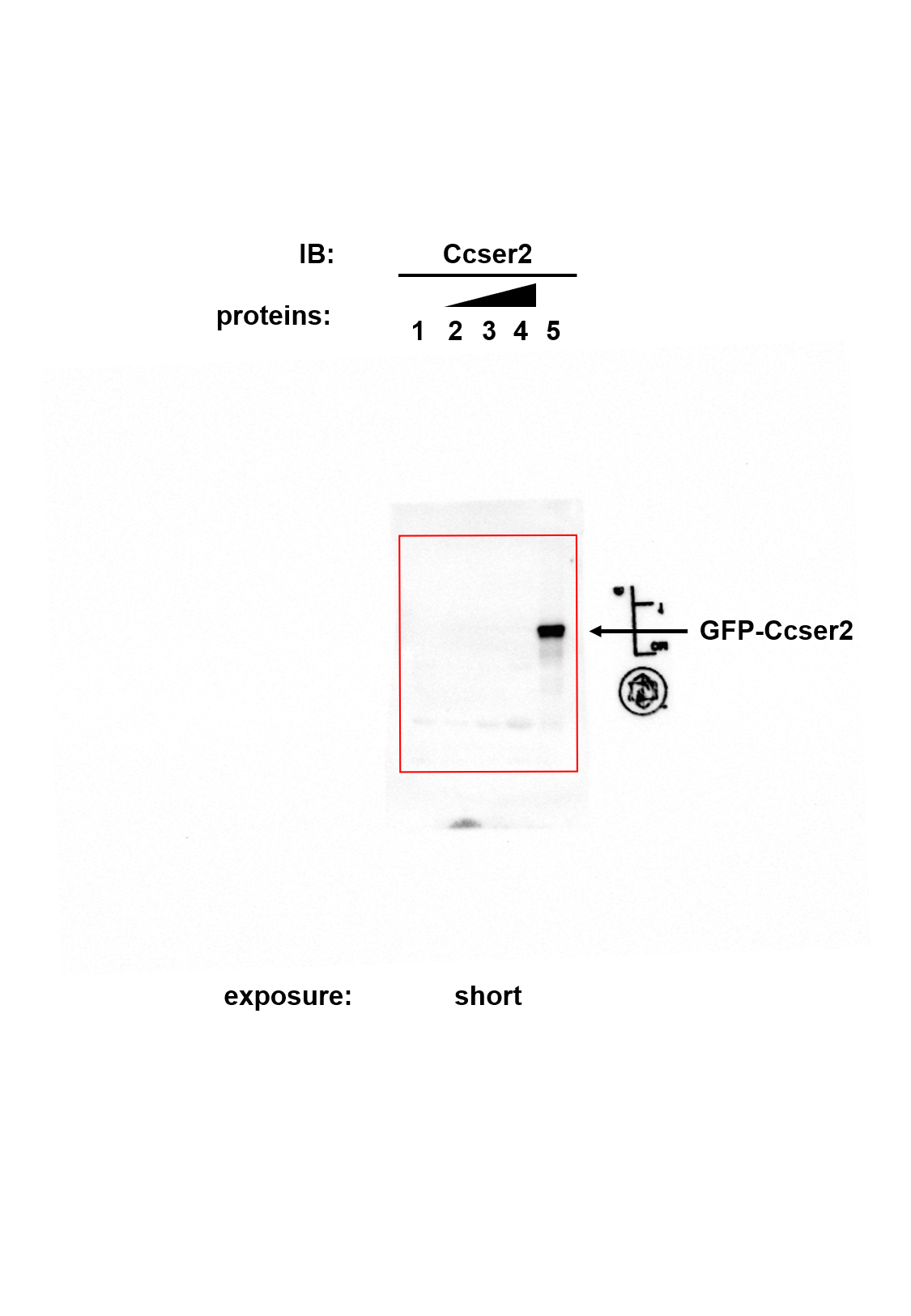

Supplement: Supplementary file 10 — Additional file 10: Figure S9. Full-size uncropped image of the immunoblot presented in Figure S8 (left blot). The red box indicates where the image was cropped to be presented in the figure. The band of GFP-Ccser2 in lane 5 is indicated by the arrow. [file 13104_2023_6475_MOESM10_ESM.tif]

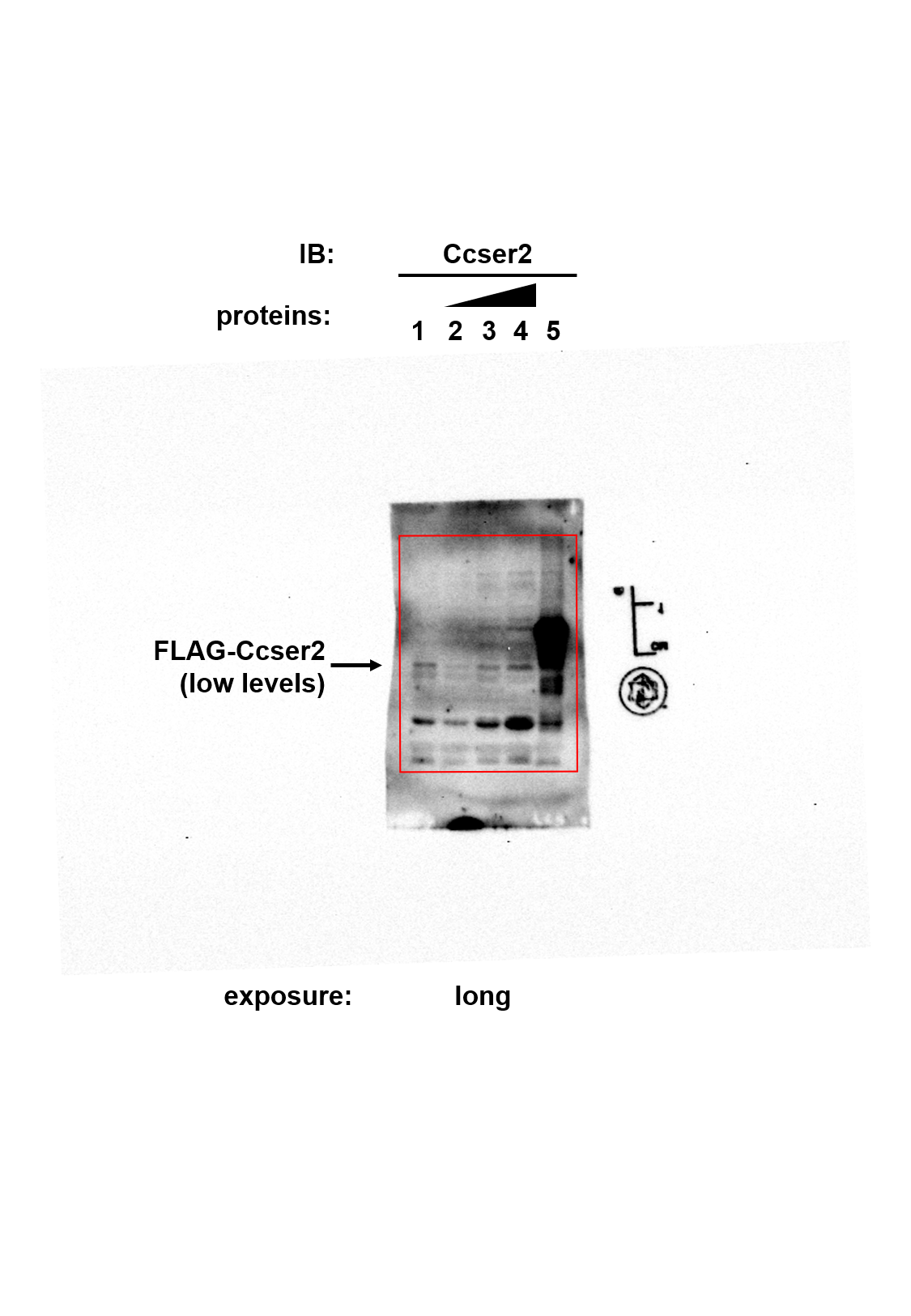

Supplement: Supplementary file 11 — Additional file 11: Figure S10. Full-size uncropped image of the immunoblot presented in Figure S8 (middle blot). The red box indicates where the image was cropped to be presented in the figure. The band of FLAG-Ccser2 (low levels) in lane 1 is indicated by the arrow. [file 13104_2023_6475_MOESM11_ESM.tif]

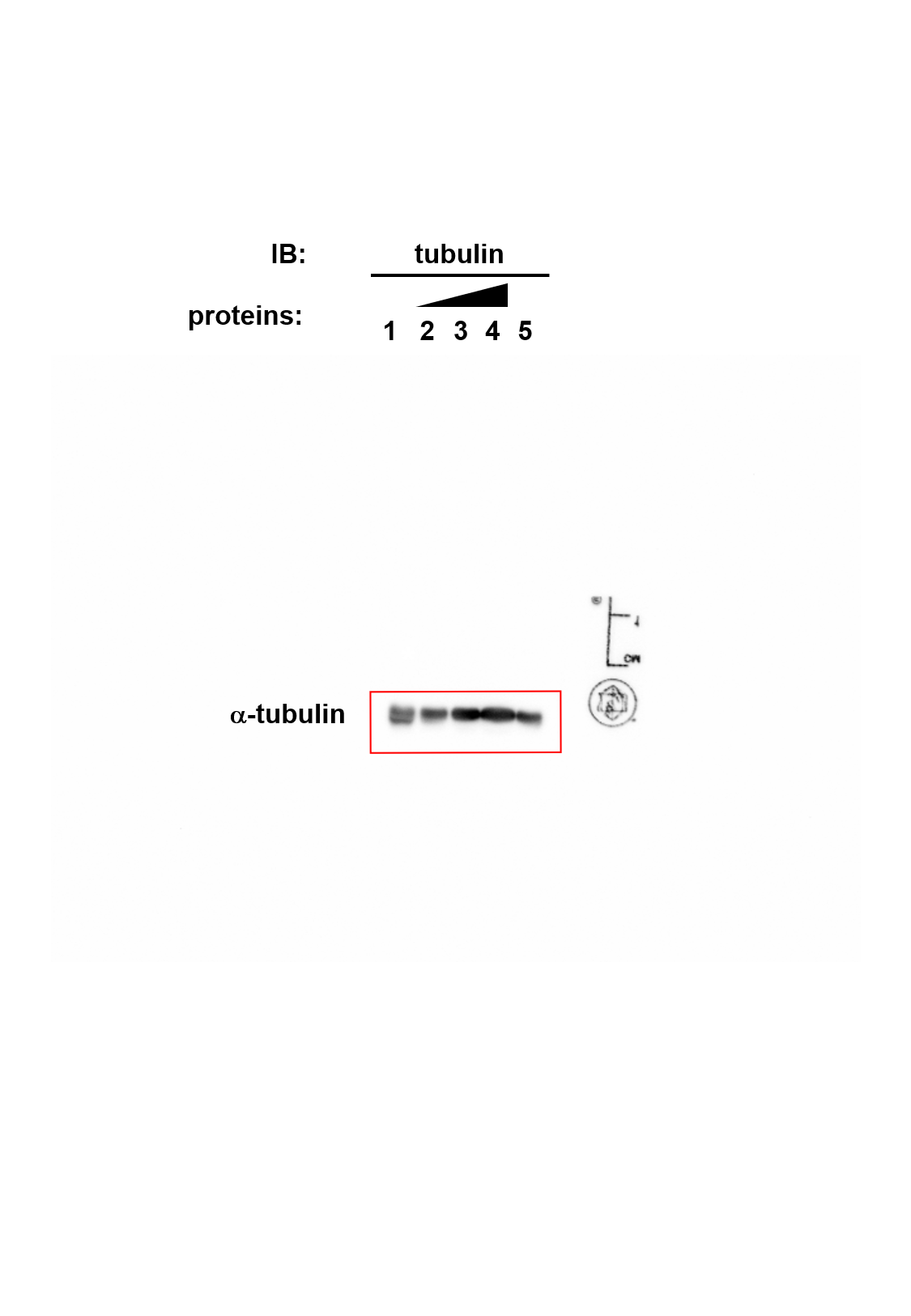

Supplement: Supplementary file 12 — Additional file 12: Figure S11. Full-size uncropped image of the immunoblot presented in Figure S8 (right blot). The red box indicates the bands in the figure; α-tubulin. [file 13104_2023_6475_MOESM12_ESM.tif]

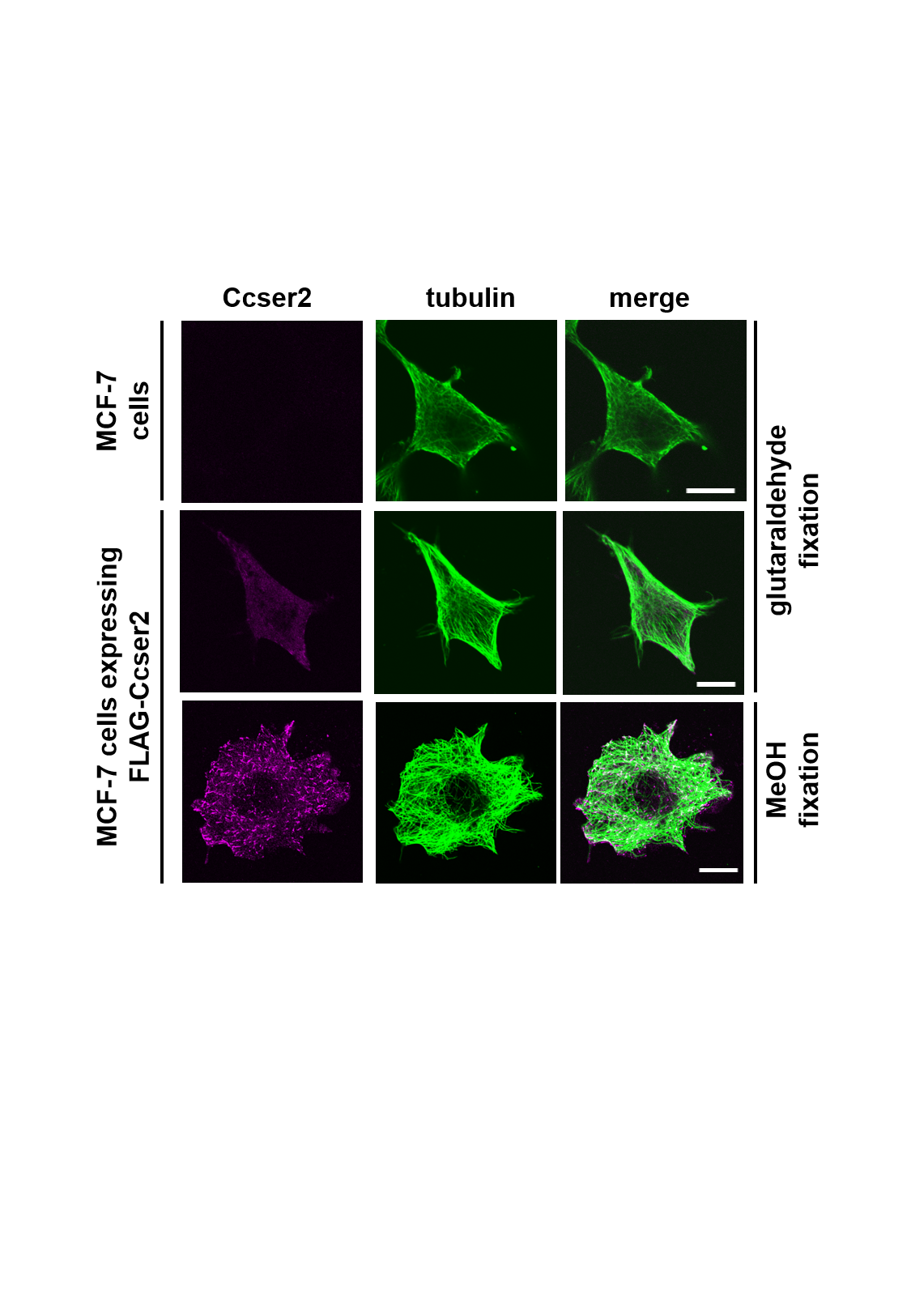

Supplement: Supplementary file 13 — Additional file 13: Figure S12. Immunocytochemical detection of human Ccser2 after glutaraldehyde fixation. MCF-7 cells (top panels) and MCF-7 cells transiently expressing FLAG-human Ccser2 (middle and bottom panels) were fixed with glutaraldehyde (top and middle panels) or cold methanol (bottom panels). These fixed cells were then subjected to double-immunostaining with antibodies against human Ccser2 and α-tubulin. Bars, 10 μm. [file 13104_2023_6475_MOESM13_ESM.tif]
